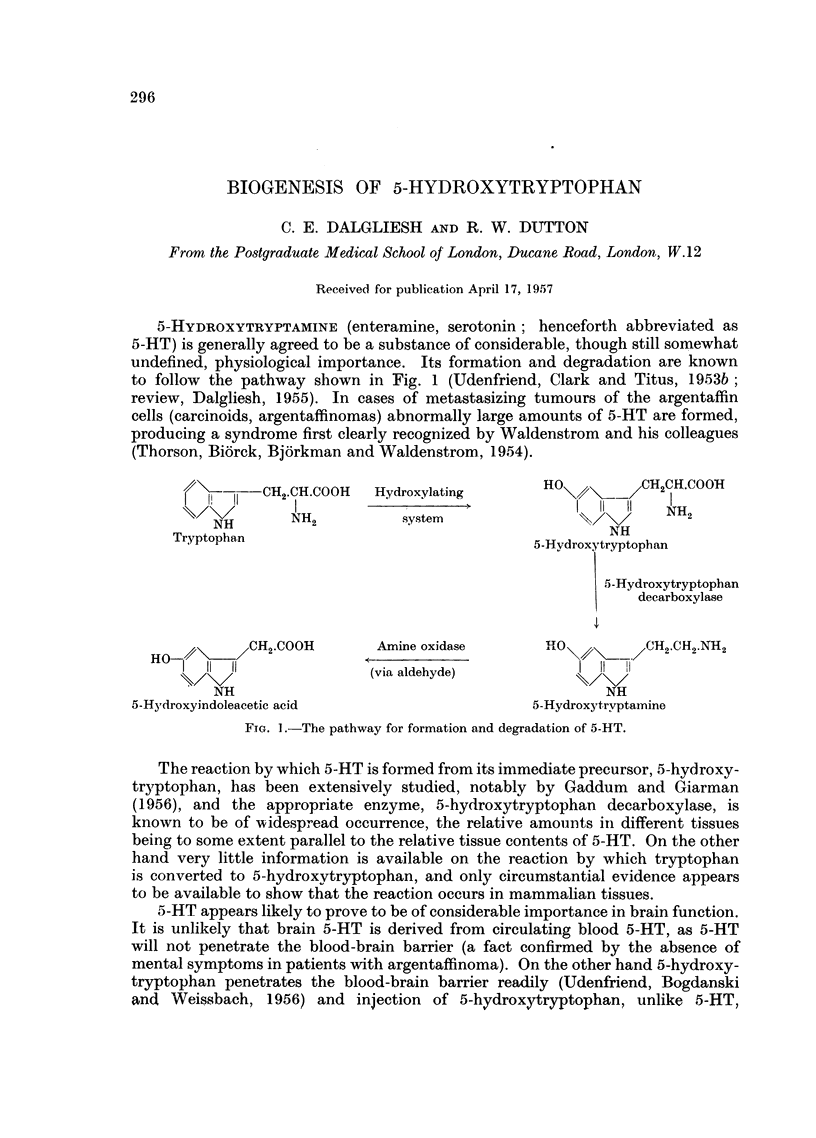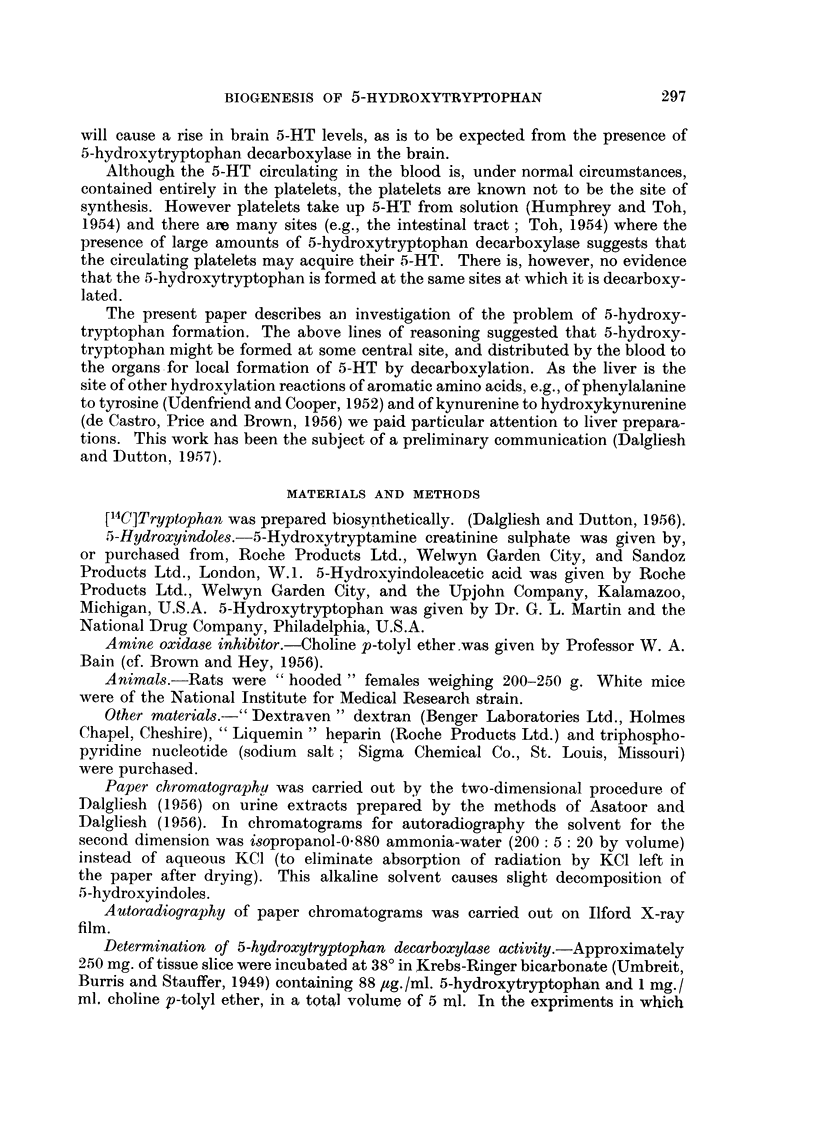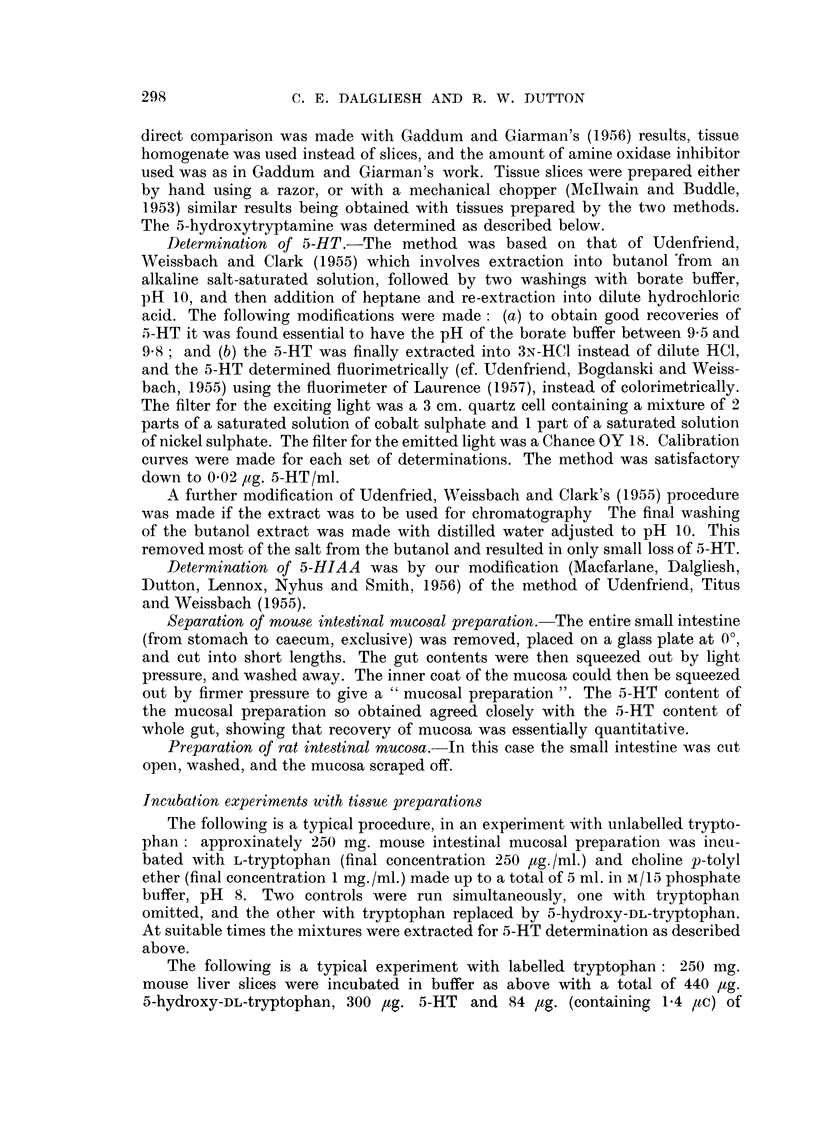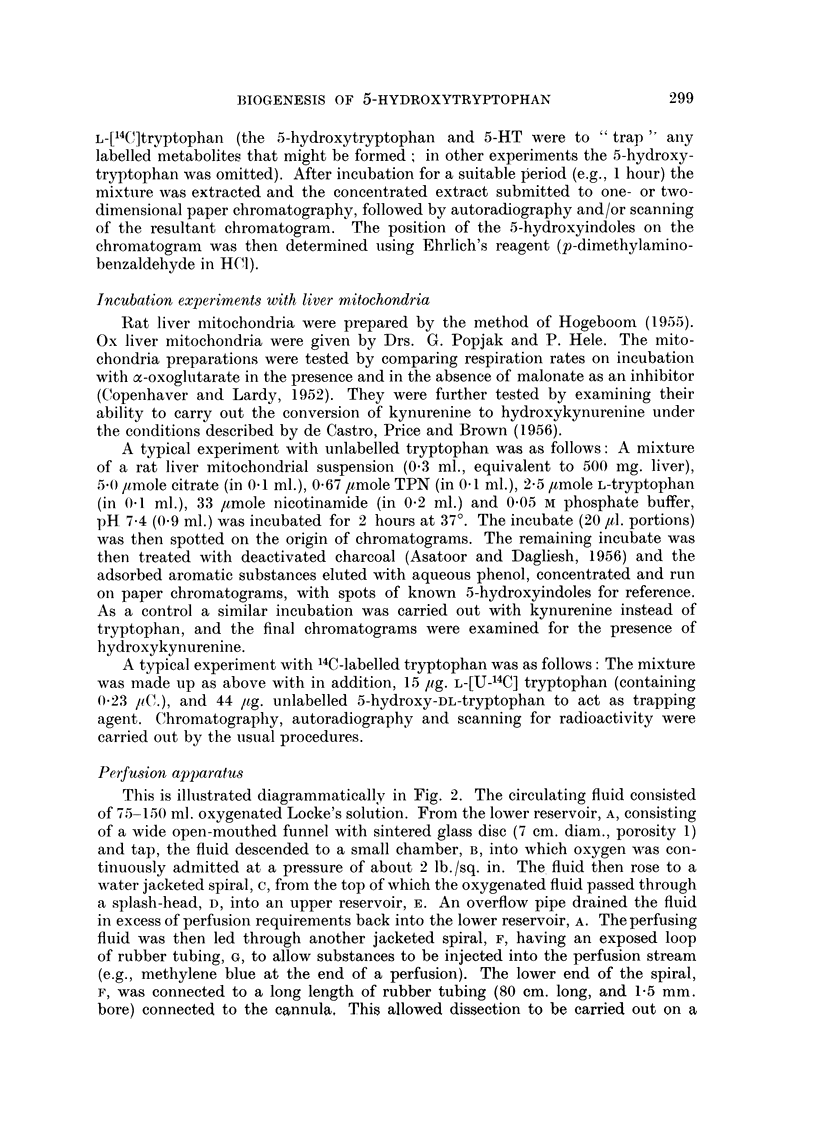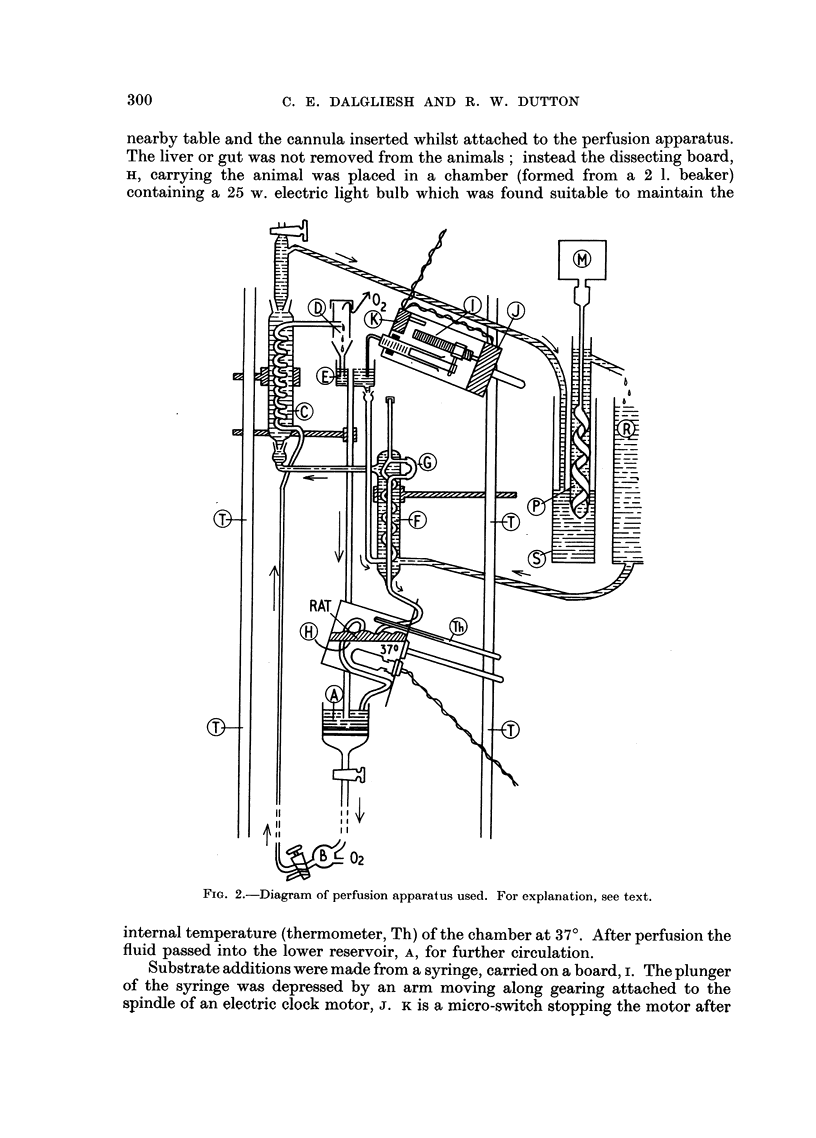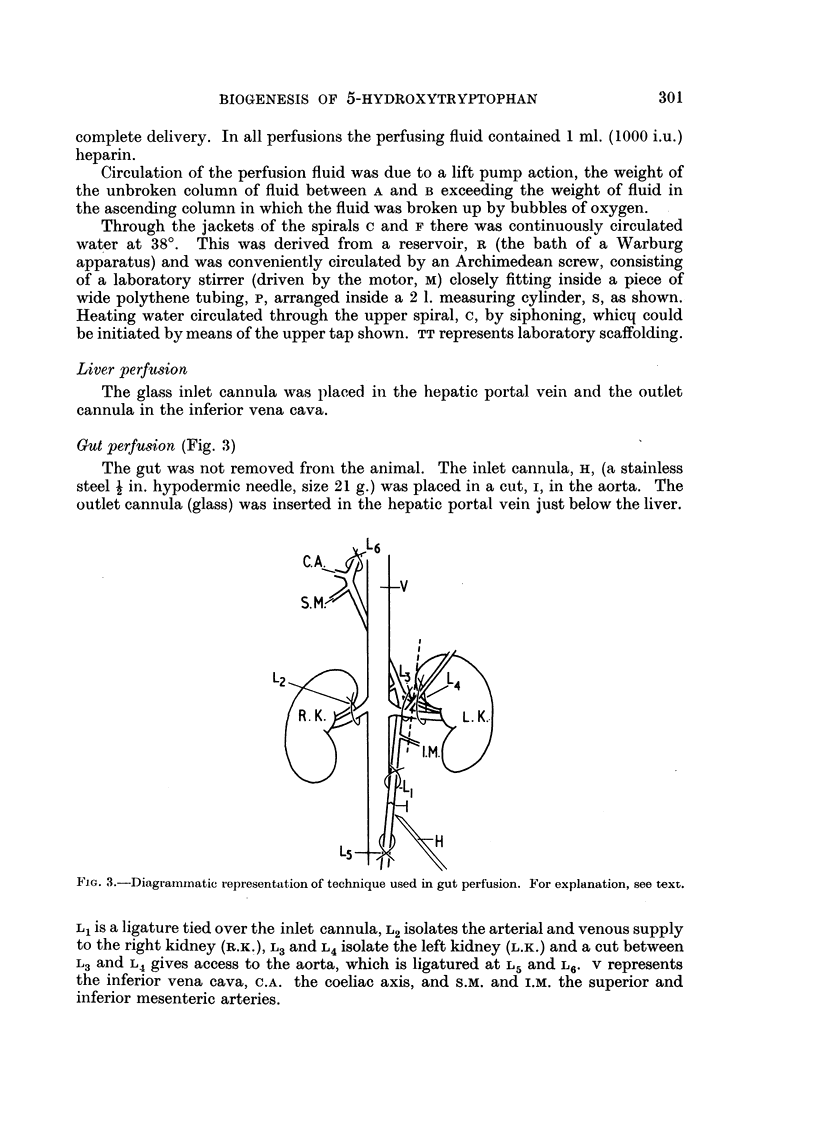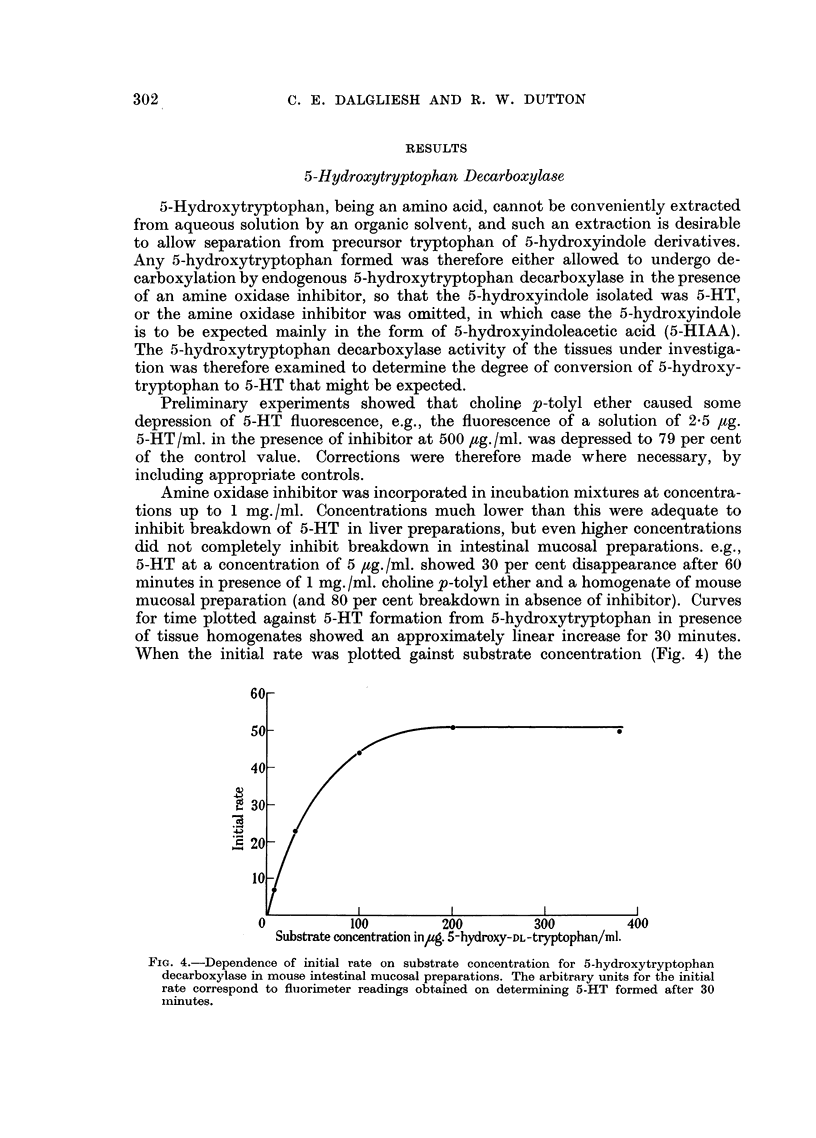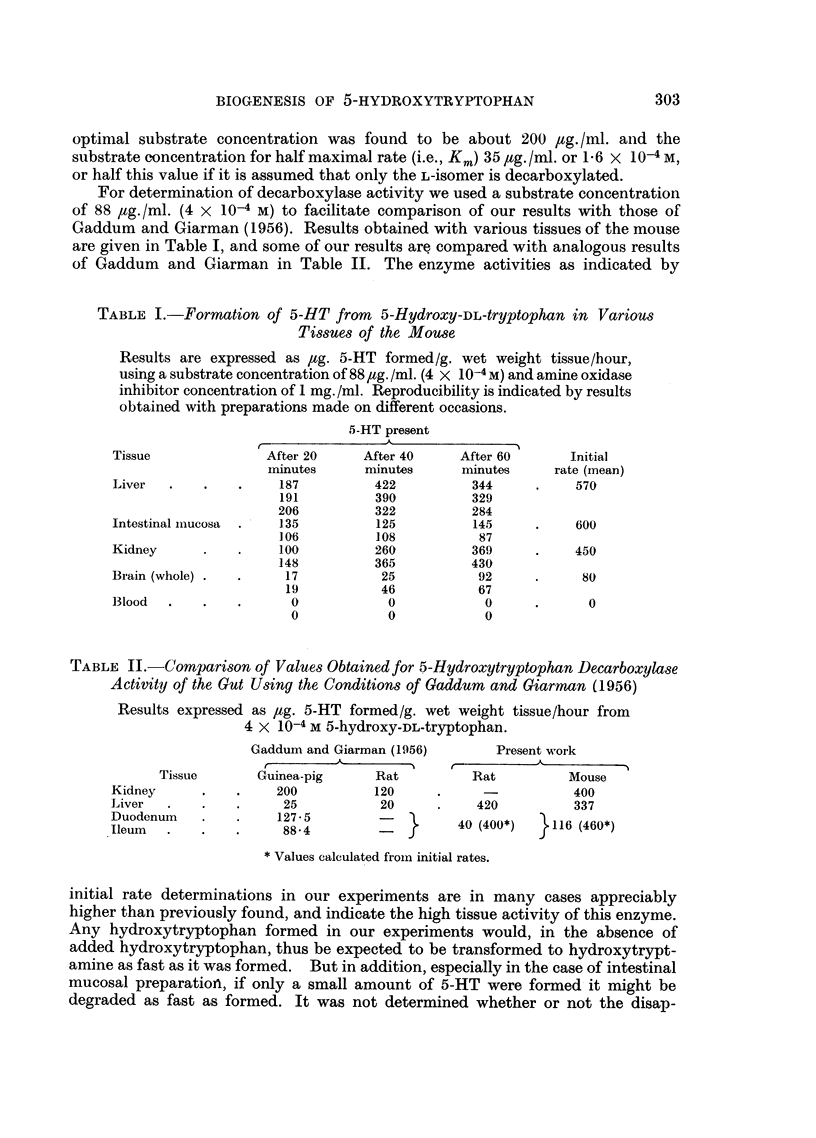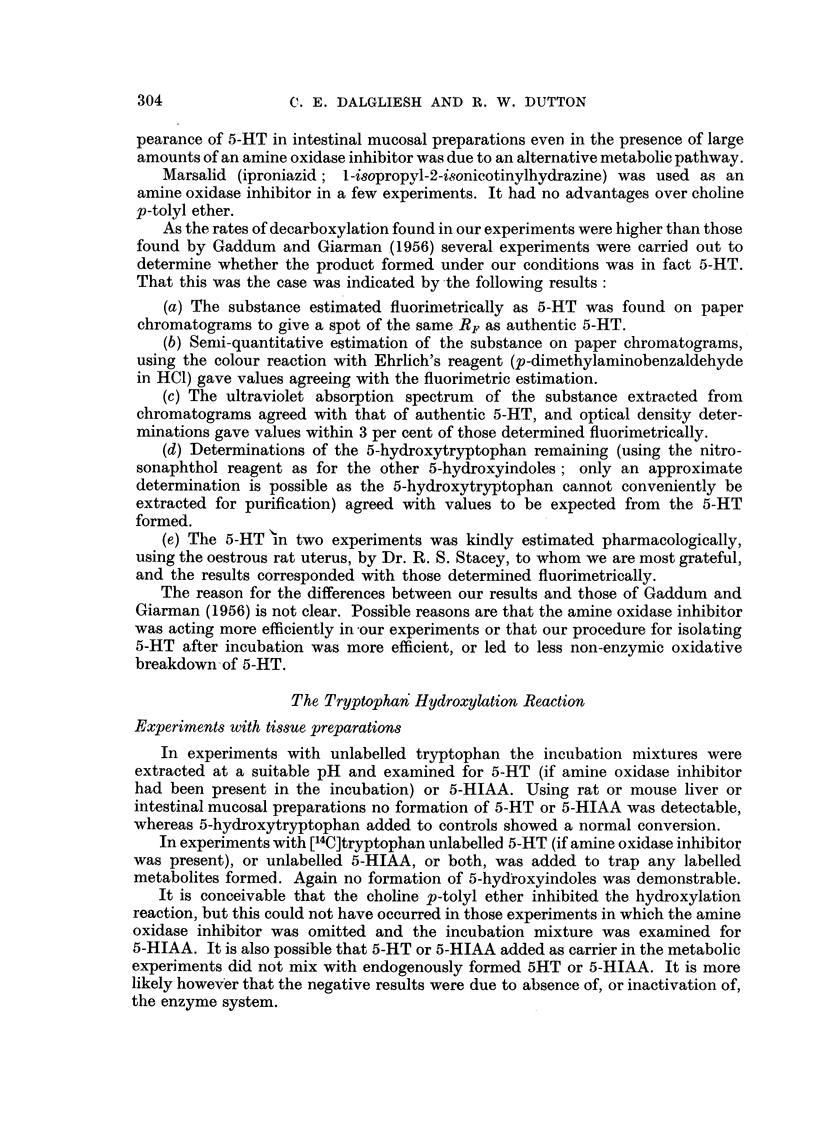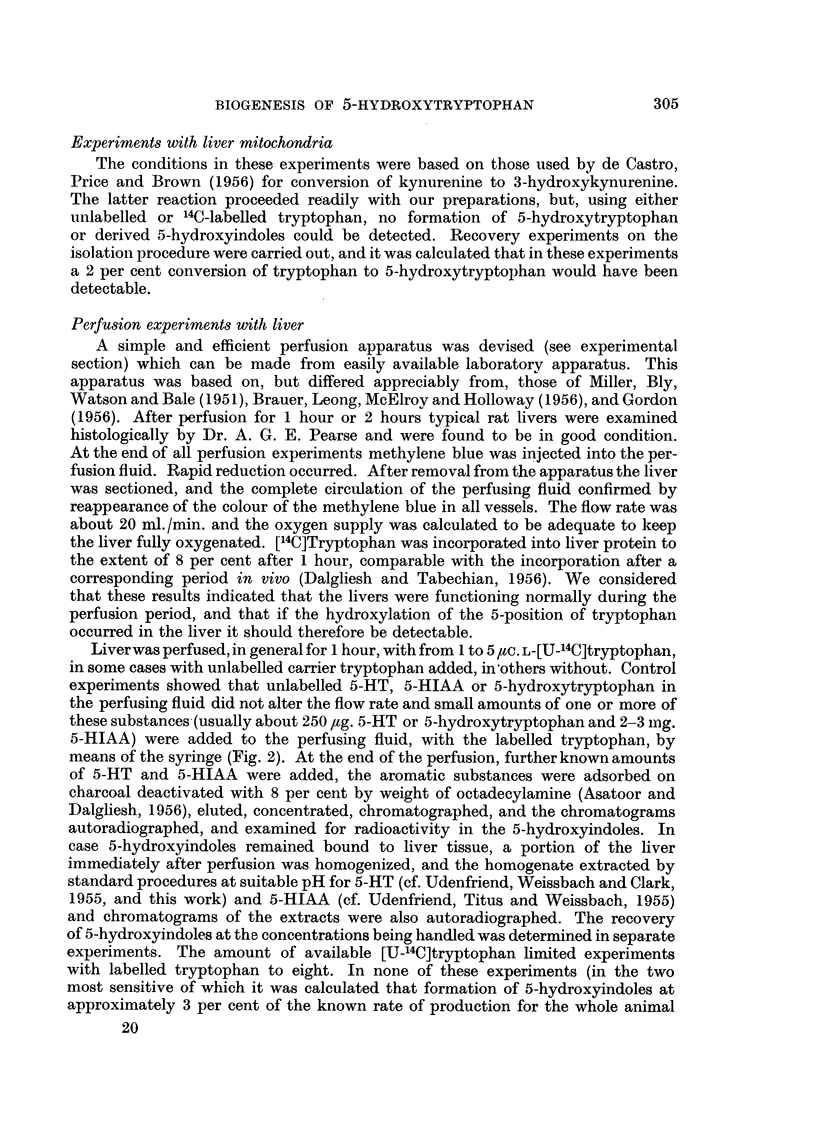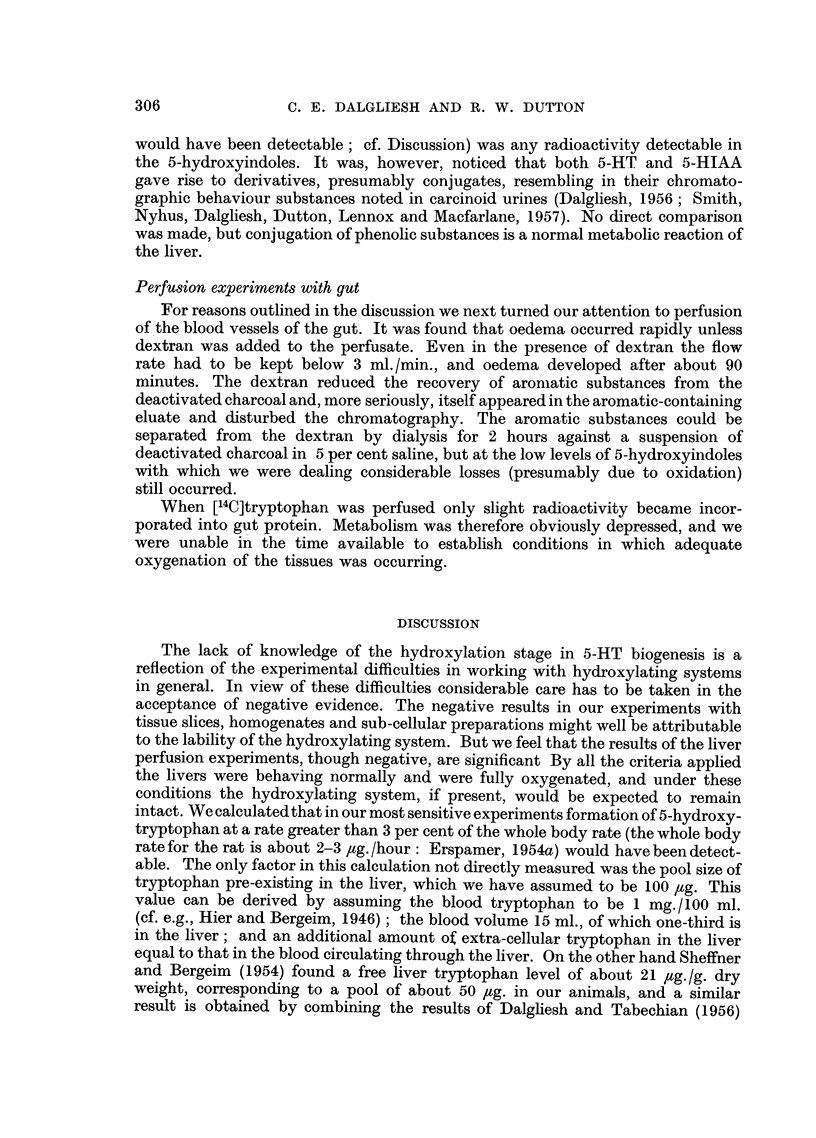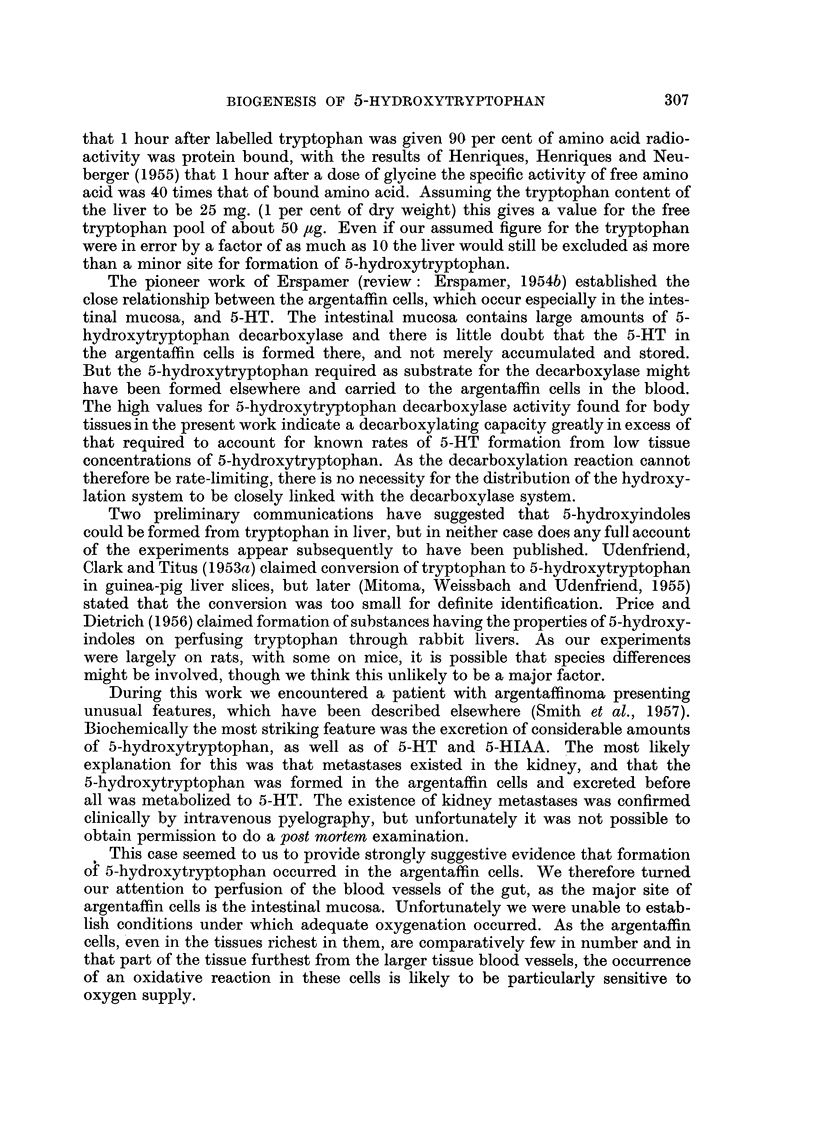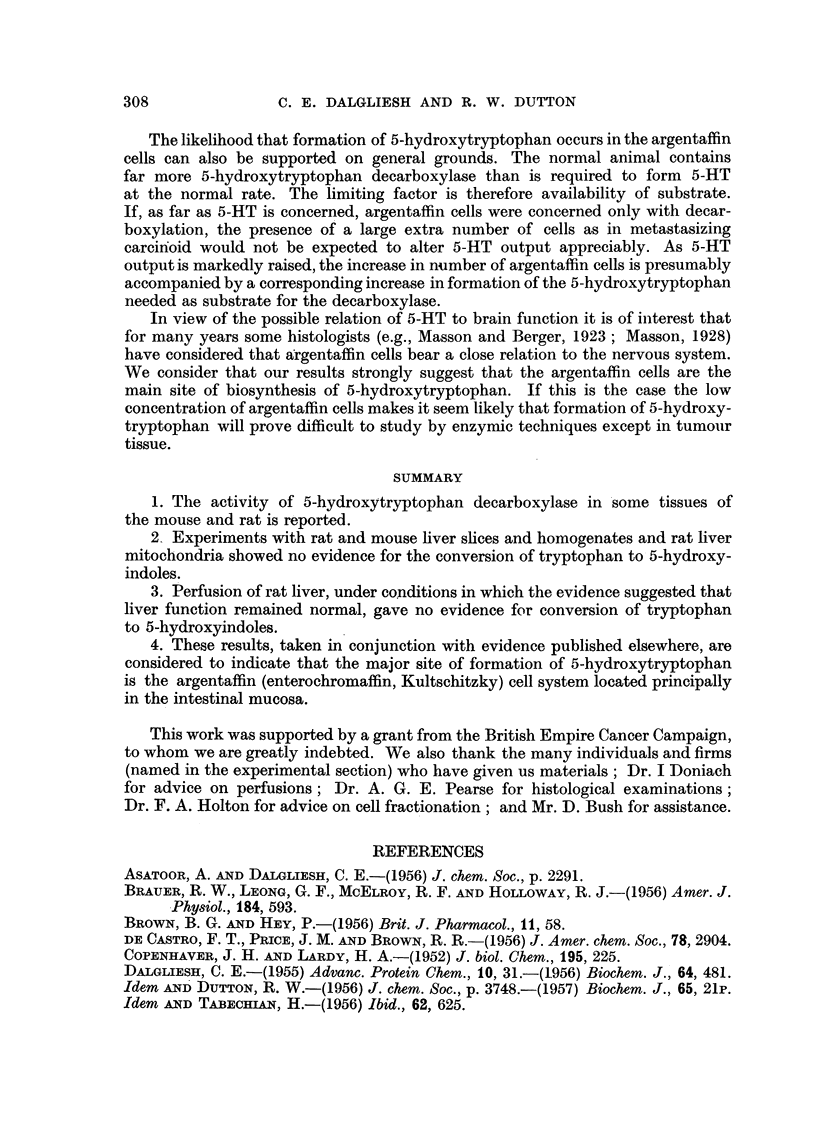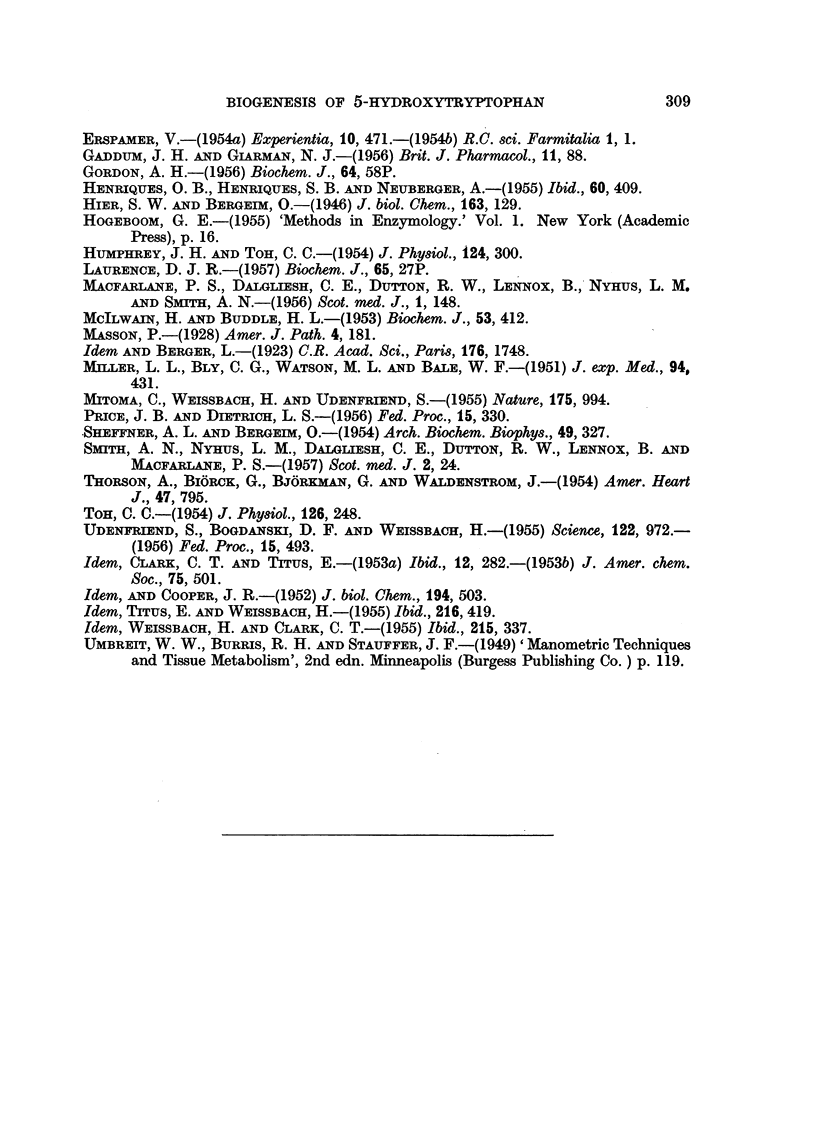# Biogenesis of 5-Hydroxytryptophan

**DOI:** 10.1038/bjc.1957.38

**Published:** 1957-06

**Authors:** C. E. Dalgliesh, R. W. Dutton


### BIOGENESIS OF 5-HYDROXYTRYPTOPHAN

C. E. DALGLIESH AND R. W. DUTTON

*From the Postgraduate Medical School of London, Ducane Road, London, W.*12

Received for publication April 17, 1957

5-HYDROXYTRYPTAMINE (enteramine, serotonin ; henceforth abbreviated as 5-HT) is generally agreed to be a substance of considerable, though still somewhat undefined, physiological importance. Its formation and degradation are known to follow the pathway shown in Fig. 1 (Udenfriend, Clark and Titus, 1953*b* ; review, Dalgliesh, 1955). In cases of metastasizing tumours of the argentaffin cells (carcinoids, argentaffinomas) abnormally large amounts of 5-HT are formed, producing a syndrome first clearly recognized by Waldenstrom and his colleagues (Thorson, Biörck, Björkman and Waldenstrom, 1954).

FIG. 1.—The pathway for formation and degradation of 5-HT.

The reaction by which 5-HT is formed from its immediate precursor, 5-hydroxytryptophan, has been extensively studied, notably by Gaddum and Giarman (1956), and the appropriate enzyme, 5-hydroxytryptophan decarboxylase, is known to be of widespread occurrence, the relative amounts in different tissues being to some extent parallel to the relative tissue contents of 5-HT. On the other hand very little information is available on the reaction by which tryptophan is converted to 5-hydroxytryptophan, and only circumstantial evidence appears to be available to show that the reaction occurs in mammalian tissues.

5-HT appears likely to prove to be of considerable importance in brain function. It is unlikely that brain 5-HT is derived from circulating blood 5-HT, as 5-HT will not penetrate the blood-brain barrier (a fact confirmed by the absence of mental symptoms in patients with argentaffinoma). On the other hand 5-hydroxytryptophan penetrates the blood-brain barrier readily (Udenfriend, Bogdanski and Weissbach, 1956) and injection of 5-hydroxytryptophan, unlike 5-HT, will cause a rise in brain 5-HT levels, as is to be expected from the presence of 5-hydroxytryptophan decarboxylase in the brain.

Although the 5-HT circulating in the blood is, under normal circumstances, contained entirely in the platelets, the platelets are known not to be the site of synthesis. However platelets take up 5-HT from solution (Humphrey and Toh, 1954) and there are many sites (e.g., the intestinal tract ; Toh, 1954) where the presence of large amounts of 5-hydroxytryptophan decarboxylase suggests that the circulating platelets may acquire their 5-HT. There is, however, no evidence that the 5-hydroxytryptophan is formed at the same sites at which it is decarboxylated.

The present paper describes an investigation of the problem of 5-hydroxytryptophan formation. The above lines of reasoning suggested that 5-hydroxytryptophan might be formed at some central site, and distributed by the blood to the organs for local formation of 5-HT by decarboxylation. As the liver is the site of other hydroxylation reactions of aromatic amino acids, e.g., of phenylalanine to tyrosine (Udenfriend and Cooper, 1952) and of kynurenine to hydroxykynurenine (de Castro, Price and Brown, 1956) we paid particular attention to liver preparations. This work has been the subject of a preliminary communication (Dalgliesh and Dutton, 1957).

### MATERIALS AND METHODS

*[$^{14}C$]Tryptophan* was prepared biosynthetically. (Dalgliesh and Dutton, 1956).

*5-Hydroxyindoles.*—5-Hydroxytryptamine creatinine sulphate was given by, or purchased from, Roche Products Ltd., Welwyn Garden City, and Sandoz Products Ltd., London, W.1. 5-Hydroxyindoleacetic acid was given by Roche Products Ltd., Welwyn Garden City, and the Upjohn Company, Kalamazoo, Michigan, U.S.A. 5-Hydroxytryptophan was given by Dr. G. L. Martin and the National Drug Company, Philadelphia, U.S.A.

*Amine oxidase inhibitor.*—Choline *p*-tolyl ether was given by Professor W. A. Bain (cf. Brown and Hey, 1956).

*Animals.*—Rats were " hooded " females weighing 200–250 g. White mice were of the National Institute for Medical Research strain.

*Other materials.*—" Dextraven " dextran (Benger Laboratories Ltd., Holmes Chapel, Cheshire), " Liquemin " heparin (Roche Products Ltd.) and triphosphopyridine nucleotide (sodium salt ; Sigma Chemical Co., St. Louis, Missouri) were purchased.

*Paper chromatography* was carried out by the two-dimensional procedure of Dalgliesh (1956) on urine extracts prepared by the methods of Asatoor and Dalgliesh (1956). In chromatograms for autoradiography the solvent for the second dimension was *iso*propanol-0·880 ammonia-water (200 : 5 : 20 by volume) instead of aqueous KCl (to eliminate absorption of radiation by KCl left in the paper after drying). This alkaline solvent causes slight decomposition of 5-hydroxyindoles.

*Autoradiography* of paper chromatograms was carried out on Ilford X-ray film.

*Determination of 5-hydroxytryptophan decarboxylase activity.*—Approximately 250 mg. of tissue slice were incubated at 38° in Krebs-Ringer bicarbonate (Umbreit, Burris and Stauffer, 1949) containing 88 μg./ml. 5-hydroxytryptophan and 1 mg./ml. choline *p*-tolyl ether, in a total volume of 5 ml. In the expriments in which direct comparison was made with Gaddum and Giarman's (1956) results, tissue homogenate was used instead of slices, and the amount of amine oxidase inhibitor used was as in Gaddum and Giarman's work. Tissue slices were prepared either by hand using a razor, or with a mechanical chopper (McIlwain and Buddle, 1953) similar results being obtained with tissues prepared by the two methods. The 5-hydroxytryptamine was determined as described below.

*Determination of 5-HT*.—The method was based on that of Udenfriend, Weissbach and Clark (1955) which involves extraction into butanol from an alkaline salt-saturated solution, followed by two washings with borate buffer, pH 10, and then addition of heptane and re-extraction into dilute hydrochloric acid. The following modifications were made : (*a*) to obtain good recoveries of 5-HT it was found essential to have the pH of the borate buffer between 9·5 and 9·8 ; and (*b*) the 5-HT was finally extracted into 3N-HCl instead of dilute HCl, and the 5-HT determined fluorimetrically (cf. Udenfriend, Bogdanski and Weissbach, 1955) using the fluorimeter of Laurence (1957), instead of colorimetrically. The filter for the exciting light was a 3 cm. quartz cell containing a mixture of 2 parts of a saturated solution of cobalt sulphate and 1 part of a saturated solution of nickel sulphate. The filter for the emitted light was a Chance OY 18. Calibration curves were made for each set of determinations. The method was satisfactory down to 0·02 $\mu$g. 5-HT/ml.

A further modification of Udenfried, Weissbach and Clark's (1955) procedure was made if the extract was to be used for chromatography The final washing of the butanol extract was made with distilled water adjusted to pH 10. This removed most of the salt from the butanol and resulted in only small loss of 5-HT.

*Determination of 5-HIAA* was by our modification (Macfarlane, Dalgliesh, Dutton, Lennox, Nyhus and Smith, 1956) of the method of Udenfriend, Titus and Weissbach (1955).

*Separation of mouse intestinal mucosal preparation*.—The entire small intestine (from stomach to caecum, exclusive) was removed, placed on a glass plate at 0°, and cut into short lengths. The gut contents were then squeezed out by light pressure, and washed away. The inner coat of the mucosa could then be squeezed out by firmer pressure to give a " mucosal preparation ". The 5-HT content of the mucosal preparation so obtained agreed closely with the 5-HT content of whole gut, showing that recovery of mucosa was essentially quantitative.

*Preparation of rat intestinal mucosa*.—In this case the small intestine was cut open, washed, and the mucosa scraped off.

*Incubation experiments with tissue preparations*

The following is a typical procedure, in an experiment with unlabelled tryptophan : approxinately 250 mg. mouse intestinal mucosal preparation was incubated with L-tryptophan (final concentration 250 $\mu$g./ml.) and choline *p*-tolyl ether (final concentration 1 mg./ml.) made up to a total of 5 ml. in M/15 phosphate buffer, pH 8. Two controls were run simultaneously, one with tryptophan omitted, and the other with tryptophan replaced by 5-hydroxy-DL-tryptophan. At suitable times the mixtures were extracted for 5-HT determination as described above.

The following is a typical experiment with labelled tryptophan : 250 mg. mouse liver slices were incubated in buffer as above with a total of 440 $\mu$g. 5-hydroxy-DL-tryptophan, 300 $\mu$g. 5-HT and 84 $\mu$g. (containing 1·4 $\mu$c) of

L-[$^{14}$C]tryptophan (the 5-hydroxytryptophan and 5-HT were to "trap" any labelled metabolites that might be formed; in other experiments the 5-hydroxytryptophan was omitted). After incubation for a suitable period (e.g., 1 hour) the mixture was extracted and the concentrated extract submitted to one- or two-dimensional paper chromatography, followed by autoradiography and/or scanning of the resultant chromatogram. The position of the 5-hydroxyindoles on the chromatogram was then determined using Ehrlich's reagent (*p*-dimethylaminobenzaldehyde in HCl).

*Incubation experiments with liver mitochondria*

Rat liver mitochondria were prepared by the method of Hogeboom (1955). Ox liver mitochondria were given by Drs. G. Popjak and P. Hele. The mitochondria preparations were tested by comparing respiration rates on incubation with $\alpha$-oxoglutarate in the presence and in the absence of malonate as an inhibitor (Copenhaver and Lardy, 1952). They were further tested by examining their ability to carry out the conversion of kynurenine to hydroxykynurenine under the conditions described by de Castro, Price and Brown (1956).

A typical experiment with unlabelled tryptophan was as follows: A mixture of a rat liver mitochondrial suspension (0·3 ml., equivalent to 500 mg. liver), 5·0 $\mu$mole citrate (in 0·1 ml.), 0·67 $\mu$mole TPN (in 0·1 ml.), 2·5 $\mu$mole L-tryptophan (in 0·1 ml.), 33 $\mu$mole nicotinamide (in 0·2 ml.) and 0·05 M phosphate buffer, pH 7·4 (0·9 ml.) was incubated for 2 hours at 37°. The incubate (20 $\mu$l. portions) was then spotted on the origin of chromatograms. The remaining incubate was then treated with deactivated charcoal (Asatoor and Dagliesh, 1956) and the adsorbed aromatic substances eluted with aqueous phenol, concentrated and run on paper chromatograms, with spots of known 5-hydroxyindoles for reference. As a control a similar incubation was carried out with kynurenine instead of tryptophan, and the final chromatograms were examined for the presence of hydroxykynurenine.

A typical experiment with $^{14}$C-labelled tryptophan was as follows: The mixture was made up as above with in addition, 15 $\mu$g. L-[U-$^{14}$C] tryptophan (containing 0·23 $\mu$C.), and 44 $\mu$g. unlabelled 5-hydroxy-DL-tryptophan to act as trapping agent. Chromatography, autoradiography and scanning for radioactivity were carried out by the usual procedures.

*Perfusion apparatus*

This is illustrated diagrammatically in Fig. 2. The circulating fluid consisted of 75–150 ml. oxygenated Locke's solution. From the lower reservoir, A, consisting of a wide open-mouthed funnel with sintered glass disc (7 cm. diam., porosity 1) and tap, the fluid descended to a small chamber, B, into which oxygen was continuously admitted at a pressure of about 2 lb./sq. in. The fluid then rose to a water jacketed spiral, C, from the top of which the oxygenated fluid passed through a splash-head, D, into an upper reservoir, E. An overflow pipe drained the fluid in excess of perfusion requirements back into the lower reservoir, A. The perfusing fluid was then led through another jacketed spiral, F, having an exposed loop of rubber tubing, G, to allow substances to be injected into the perfusion stream (e.g., methylene blue at the end of a perfusion). The lower end of the spiral, F, was connected to a long length of rubber tubing (80 cm. long, and 1·5 mm. bore) connected to the cannula. This allowed dissection to be carried out on a nearby table and the cannula inserted whilst attached to the perfusion apparatus. The liver or gut was not removed from the animals ; instead the dissecting board, H, carrying the animal was placed in a chamber (formed from a 2 l. beaker) containing a 25 w. electric light bulb which was found suitable to maintain the

FIG. 2.—Diagram of perfusion apparatus used. For explanation, see text.

internal temperature (thermometer, Th) of the chamber at 37°. After perfusion the fluid passed into the lower reservoir, A, for further circulation.

Substrate additions were made from a syringe, carried on a board, I. The plunger of the syringe was depressed by an arm moving along gearing attached to the spindle of an electric clock motor, J. K is a micro-switch stopping the motor after complete delivery. In all perfusions the perfusing fluid contained 1 ml. (1000 i.u.) heparin.

Circulation of the perfusion fluid was due to a lift pump action, the weight of the unbroken column of fluid between A and B exceeding the weight of fluid in the ascending column in which the fluid was broken up by bubbles of oxygen.

Through the jackets of the spirals C and F there was continuously circulated water at 38°. This was derived from a reservoir, R (the bath of a Warburg apparatus) and was conveniently circulated by an Archimedean screw, consisting of a laboratory stirrer (driven by the motor, M) closely fitting inside a piece of wide polythene tubing, P, arranged inside a 2 l. measuring cylinder, S, as shown. Heating water circulated through the upper spiral, C, by siphoning, whicq could be initiated by means of the upper tap shown. TT represents laboratory scaffolding.

*Liver perfusion*

The glass inlet cannula was placed in the hepatic portal vein and the outlet cannula in the inferior vena cava.

*Gut perfusion* (Fig. 3)

The gut was not removed from the animal. The inlet cannula, H, (a stainless steel ½ in. hypodermic needle, size 21 g.) was placed in a cut, I, in the aorta. The outlet cannula (glass) was inserted in the hepatic portal vein just below the liver.

FIG. 3.—Diagrammatic representation of technique used in gut perfusion. For explanation, see text.

$L_1$ is a ligature tied over the inlet cannula, $L_2$ isolates the arterial and venous supply to the right kidney (R.K.), $L_3$ and $L_4$ isolate the left kidney (L.K.) and a cut between $L_3$ and $L_4$ gives access to the aorta, which is ligatured at $L_5$ and $L_6$. V represents the inferior vena cava, C.A. the coeliac axis, and S.M. and I.M. the superior and inferior mesenteric arteries.

### RESULTS

### 5-*Hydroxytryptophan Decarboxylase*

5-Hydroxytryptophan, being an amino acid, cannot be conveniently extracted from aqueous solution by an organic solvent, and such an extraction is desirable to allow separation from precursor tryptophan of 5-hydroxyindole derivatives. Any 5-hydroxytryptophan formed was therefore either allowed to undergo decarboxylation by endogenous 5-hydroxytryptophan decarboxylase in the presence of an amine oxidase inhibitor, so that the 5-hydroxyindole isolated was 5-HT, or the amine oxidase inhibitor was omitted, in which case the 5-hydroxyindole is to be expected mainly in the form of 5-hydroxyindoleacetic acid (5-HIAA). The 5-hydroxytryptophan decarboxylase activity of the tissues under investigation was therefore examined to determine the degree of conversion of 5-hydroxytryptophan to 5-HT that might be expected.

Preliminary experiments showed that choline *p*-tolyl ether caused some depression of 5-HT fluorescence, e.g., the fluorescence of a solution of 2·5 μg. 5-HT/ml. in the presence of inhibitor at 500 μg./ml. was depressed to 79 per cent of the control value. Corrections were therefore made where necessary, by including appropriate controls.

Amine oxidase inhibitor was incorporated in incubation mixtures at concentrations up to 1 mg./ml. Concentrations much lower than this were adequate to inhibit breakdown of 5-HT in liver preparations, but even higher concentrations did not completely inhibit breakdown in intestinal mucosal preparations. e.g., 5-HT at a concentration of 5 μg./ml. showed 30 per cent disappearance after 60 minutes in presence of 1 mg./ml. choline *p*-tolyl ether and a homogenate of mouse mucosal preparation (and 80 per cent breakdown in absence of inhibitor). Curves for time plotted against 5-HT formation from 5-hydroxytryptophan in presence of tissue homogenates showed an approximately linear increase for 30 minutes. When the initial rate was plotted gainst substrate concentration (Fig. 4) the

FIG. 4.—Dependence of initial rate on substrate concentration for 5-hydroxytryptophan decarboxylase in mouse intestinal mucosal preparations. The arbitrary units for the initial rate correspond to fluorimeter readings obtained on determining 5-HT formed after 30 minutes.

optimal substrate concentration was found to be about 200 μg./ml. and the substrate concentration for half maximal rate (i.e., $K_m$) 35 μg./ml. or $1 \cdot 6 \times 10^{-4}$ M, or half this value if it is assumed that only the L-isomer is decarboxylated.

For determination of decarboxylase activity we used a substrate concentration of 88 μg./ml. ($4 \times 10^{-4}$ M) to facilitate comparison of our results with those of Gaddum and Giarman (1956). Results obtained with various tissues of the mouse are given in Table I, and some of our results are compared with analogous results of Gaddum and Giarman in Table II. The enzyme activities as indicated by

TABLE I.—*Formation of 5-HT from 5-Hydroxy-DL-tryptophan in Various Tissues of the Mouse*

Results are expressed as μg. 5-HT formed/g. wet weight tissue/hour, using a substrate concentration of 88 μg./ml. ($4 \times 10^{-4}$ M) and amine oxidase inhibitor concentration of 1 mg./ml. Reproducibility is indicated by results obtained with preparations made on different occasions.

| Tissue | 5-HT present | | | Initial rate (mean) |
|---|---|---|---|---|
| | After 20 minutes | After 40 minutes | After 60 minutes | |
| Liver | 187 | 422 | 344 | 570 |
| | 191 | 390 | 329 | |
| | 206 | 322 | 284 | |
| Intestinal mucosa | 135 | 125 | 145 | 600 |
| | 106 | 108 | 87 | |
| Kidney | 100 | 260 | 369 | 450 |
| | 148 | 365 | 430 | |
| Brain (whole) | 17 | 25 | 92 | 80 |
| | 19 | 46 | 67 | |
| Blood | 0 | 0 | 0 | 0 |
| | 0 | 0 | 0 | |

TABLE II.—*Comparison of Values Obtained for 5-Hydroxytryptophan Decarboxylase Activity of the Gut Using the Conditions of Gaddum and Giarman* (1956)

Results expressed as μg. 5-HT formed/g. wet weight tissue/hour from $4 \times 10^{-4}$ M 5-hydroxy-DL-tryptophan.

| | Gaddum and Giarman (1956) | | Present work | |
|---|---|---|---|---|
| Tissue | Guinea-pig | Rat | Rat | Mouse |
| Kidney | 200 | 120 | — | 400 |
| Liver | 25 | 20 | 420 | 337 |
| Duodenum | 127·5 | — | 40 (400*) | 116 (460*) |
| Ileum | 88·4 | — | | |

* Values calculated from initial rates.

initial rate determinations in our experiments are in many cases appreciably higher than previously found, and indicate the high tissue activity of this enzyme. Any hydroxytryptophan formed in our experiments would, in the absence of added hydroxytryptophan, thus be expected to be transformed to hydroxytryptamine as fast as it was formed. But in addition, especially in the case of intestinal mucosal preparation, if only a small amount of 5-HT were formed it might be degraded as fast as formed. It was not determined whether or not the disappearance of 5-HT in intestinal mucosal preparations even in the presence of large amounts of an amine oxidase inhibitor was due to an alternative metabolic pathway.

Marsalid (iproniazid; 1-*iso*propyl-2-*iso*nicotinylhydrazine) was used as an amine oxidase inhibitor in a few experiments. It had no advantages over choline *p*-tolyl ether.

As the rates of decarboxylation found in our experiments were higher than those found by Gaddum and Giarman (1956) several experiments were carried out to determine whether the product formed under our conditions was in fact 5-HT. That this was the case was indicated by the following results:

(*a*) The substance estimated fluorimetrically as 5-HT was found on paper chromatograms to give a spot of the same $R_F$ as authentic 5-HT.

(*b*) Semi-quantitative estimation of the substance on paper chromatograms, using the colour reaction with Ehrlich's reagent (*p*-dimethylaminobenzaldehyde in HCl) gave values agreeing with the fluorimetric estimation.

(*c*) The ultraviolet absorption spectrum of the substance extracted from chromatograms agreed with that of authentic 5-HT, and optical density determinations gave values within 3 per cent of those determined fluorimetrically.

(*d*) Determinations of the 5-hydroxytryptophan remaining (using the nitrosonaphthol reagent as for the other 5-hydroxyindoles; only an approximate determination is possible as the 5-hydroxytryptophan cannot conveniently be extracted for purification) agreed with values to be expected from the 5-HT formed.

(*e*) The 5-HT in two experiments was kindly estimated pharmacologically, using the oestrous rat uterus, by Dr. R. S. Stacey, to whom we are most grateful, and the results corresponded with those determined fluorimetrically.

The reason for the differences between our results and those of Gaddum and Giarman (1956) is not clear. Possible reasons are that the amine oxidase inhibitor was acting more efficiently in our experiments or that our procedure for isolating 5-HT after incubation was more efficient, or led to less non-enzymic oxidative breakdown of 5-HT.

### *The Tryptophan Hydroxylation Reaction*

### *Experiments with tissue preparations*

In experiments with unlabelled tryptophan the incubation mixtures were extracted at a suitable pH and examined for 5-HT (if amine oxidase inhibitor had been present in the incubation) or 5-HIAA. Using rat or mouse liver or intestinal mucosal preparations no formation of 5-HT or 5-HIAA was detectable, whereas 5-hydroxytryptophan added to controls showed a normal conversion.

In experiments with [$^{14}$C]tryptophan unlabelled 5-HT (if amine oxidase inhibitor was present), or unlabelled 5-HIAA, or both, was added to trap any labelled metabolites formed. Again no formation of 5-hydroxyindoles was demonstrable.

It is conceivable that the choline *p*-tolyl ether inhibited the hydroxylation reaction, but this could not have occurred in those experiments in which the amine oxidase inhibitor was omitted and the incubation mixture was examined for 5-HIAA. It is also possible that 5-HT or 5-HIAA added as carrier in the metabolic experiments did not mix with endogenously formed 5HT or 5-HIAA. It is more likely however that the negative results were due to absence of, or inactivation of, the enzyme system.

*Experiments with liver mitochondria*

The conditions in these experiments were based on those used by de Castro, Price and Brown (1956) for conversion of kynurenine to 3-hydroxykynurenine. The latter reaction proceeded readily with our preparations, but, using either unlabelled or $^{14}C$-labelled tryptophan, no formation of 5-hydroxytryptophan or derived 5-hydroxyindoles could be detected. Recovery experiments on the isolation procedure were carried out, and it was calculated that in these experiments a 2 per cent conversion of tryptophan to 5-hydroxytryptophan would have been detectable.

*Perfusion experiments with liver*

A simple and efficient perfusion apparatus was devised (see experimental section) which can be made from easily available laboratory apparatus. This apparatus was based on, but differed appreciably from, those of Miller, Bly, Watson and Bale (1951), Brauer, Leong, McElroy and Holloway (1956), and Gordon (1956). After perfusion for 1 hour or 2 hours typical rat livers were examined histologically by Dr. A. G. E. Pearse and were found to be in good condition. At the end of all perfusion experiments methylene blue was injected into the perfusion fluid. Rapid reduction occurred. After removal from the apparatus the liver was sectioned, and the complete circulation of the perfusing fluid confirmed by reappearance of the colour of the methylene blue in all vessels. The flow rate was about 20 ml./min. and the oxygen supply was calculated to be adequate to keep the liver fully oxygenated. [$^{14}C$]Tryptophan was incorporated into liver protein to the extent of 8 per cent after 1 hour, comparable with the incorporation after a corresponding period *in vivo* (Dalgliesh and Tabechian, 1956). We considered that these results indicated that the livers were functioning normally during the perfusion period, and that if the hydroxylation of the 5-position of tryptophan occurred in the liver it should therefore be detectable.

Liver was perfused, in general for 1 hour, with from 1 to 5 $\mu$c. L-[U-$^{14}C$]tryptophan, in some cases with unlabelled carrier tryptophan added, in others without. Control experiments showed that unlabelled 5-HT, 5-HIAA or 5-hydroxytryptophan in the perfusing fluid did not alter the flow rate and small amounts of one or more of these substances (usually about 250 $\mu$g. 5-HT or 5-hydroxytryptophan and 2–3 mg. 5-HIAA) were added to the perfusing fluid, with the labelled tryptophan, by means of the syringe (Fig. 2). At the end of the perfusion, further known amounts of 5-HT and 5-HIAA were added, the aromatic substances were adsorbed on charcoal deactivated with 8 per cent by weight of octadecylamine (Asatoor and Dalgliesh, 1956), eluted, concentrated, chromatographed, and the chromatograms autoradiographed, and examined for radioactivity in the 5-hydroxyindoles. In case 5-hydroxyindoles remained bound to liver tissue, a portion of the liver immediately after perfusion was homogenized, and the homogenate extracted by standard procedures at suitable pH for 5-HT (cf. Udenfriend, Weissbach and Clark, 1955, and this work) and 5-HIAA (cf. Udenfriend, Titus and Weissbach, 1955) and chromatograms of the extracts were also autoradiographed. The recovery of 5-hydroxyindoles at the concentrations being handled was determined in separate experiments. The amount of available [U-$^{14}C$]tryptophan limited experiments with labelled tryptophan to eight. In none of these experiments (in the two most sensitive of which it was calculated that formation of 5-hydroxyindoles at approximately 3 per cent of the known rate of production for the whole animal would have been detectable ; cf. Discussion) was any radioactivity detectable in the 5-hydroxyindoles. It was, however, noticed that both 5-HT and 5-HIAA gave rise to derivatives, presumably conjugates, resembling in their chromatographic behaviour substances noted in carcinoid urines (Dalgliesh, 1956 ; Smith, Nyhus, Dalgliesh, Dutton, Lennox and Macfarlane, 1957). No direct comparison was made, but conjugation of phenolic substances is a normal metabolic reaction of the liver.

*Perfusion experiments with gut*

For reasons outlined in the discussion we next turned our attention to perfusion of the blood vessels of the gut. It was found that oedema occurred rapidly unless dextran was added to the perfusate. Even in the presence of dextran the flow rate had to be kept below 3 ml./min., and oedema developed after about 90 minutes. The dextran reduced the recovery of aromatic substances from the deactivated charcoal and, more seriously, itself appeared in the aromatic-containing eluate and disturbed the chromatography. The aromatic substances could be separated from the dextran by dialysis for 2 hours against a suspension of deactivated charcoal in 5 per cent saline, but at the low levels of 5-hydroxyindoles with which we were dealing considerable losses (presumably due to oxidation) still occurred.

When [$^{14}$C]tryptophan was perfused only slight radioactivity became incorporated into gut protein. Metabolism was therefore obviously depressed, and we were unable in the time available to establish conditions in which adequate oxygenation of the tissues was occurring.

### DISCUSSION

The lack of knowledge of the hydroxylation stage in 5-HT biogenesis is a reflection of the experimental difficulties in working with hydroxylating systems in general. In view of these difficulties considerable care has to be taken in the acceptance of negative evidence. The negative results in our experiments with tissue slices, homogenates and sub-cellular preparations might well be attributable to the lability of the hydroxylating system. But we feel that the results of the liver perfusion experiments, though negative, are significant By all the criteria applied the livers were behaving normally and were fully oxygenated, and under these conditions the hydroxylating system, if present, would be expected to remain intact. We calculated that in our most sensitive experiments formation of 5-hydroxytryptophan at a rate greater than 3 per cent of the whole body rate (the whole body rate for the rat is about 2–3 $\mu$g./hour : Erspamer, 1954*a*) would have been detectable. The only factor in this calculation not directly measured was the pool size of tryptophan pre-existing in the liver, which we have assumed to be 100 $\mu$g. This value can be derived by assuming the blood tryptophan to be 1 mg./100 ml. (cf. e.g., Hier and Bergeim, 1946) ; the blood volume 15 ml., of which one-third is in the liver ; and an additional amount of extra-cellular tryptophan in the liver equal to that in the blood circulating through the liver. On the other hand Sheffner and Bergeim (1954) found a free liver tryptophan level of about 21 $\mu$g./g. dry weight, corresponding to a pool of about 50 $\mu$g. in our animals, and a similar result is obtained by combining the results of Dalgliesh and Tabechian (1956)

that 1 hour after labelled tryptophan was given 90 per cent of amino acid radioactivity was protein bound, with the results of Henriques, Henriques and Neuberger (1955) that 1 hour after a dose of glycine the specific activity of free amino acid was 40 times that of bound amino acid. Assuming the tryptophan content of the liver to be 25 mg. (1 per cent of dry weight) this gives a value for the free tryptophan pool of about 50 $\mu$g. Even if our assumed figure for the tryptophan were in error by a factor of as much as 10 the liver would still be excluded as more than a minor site for formation of 5-hydroxytryptophan.

The pioneer work of Erspamer (review: Erspamer, 1954*b*) established the close relationship between the argentaffin cells, which occur especially in the intestinal mucosa, and 5-HT. The intestinal mucosa contains large amounts of 5-hydroxytryptophan decarboxylase and there is little doubt that the 5-HT in the argentaffin cells is formed there, and not merely accumulated and stored. But the 5-hydroxytryptophan required as substrate for the decarboxylase might have been formed elsewhere and carried to the argentaffin cells in the blood. The high values for 5-hydroxytryptophan decarboxylase activity found for body tissues in the present work indicate a decarboxylating capacity greatly in excess of that required to account for known rates of 5-HT formation from low tissue concentrations of 5-hydroxytryptophan. As the decarboxylation reaction cannot therefore be rate-limiting, there is no necessity for the distribution of the hydroxylation system to be closely linked with the decarboxylase system.

Two preliminary communications have suggested that 5-hydroxyindoles could be formed from tryptophan in liver, but in neither case does any full account of the experiments appear subsequently to have been published. Udenfriend, Clark and Titus (1953*a*) claimed conversion of tryptophan to 5-hydroxytryptophan in guinea-pig liver slices, but later (Mitoma, Weissbach and Udenfriend, 1955) stated that the conversion was too small for definite identification. Price and Dietrich (1956) claimed formation of substances having the properties of 5-hydroxyindoles on perfusing tryptophan through rabbit livers. As our experiments were largely on rats, with some on mice, it is possible that species differences might be involved, though we think this unlikely to be a major factor.

During this work we encountered a patient with argentaffinoma presenting unusual features, which have been described elsewhere (Smith *et al.*, 1957). Biochemically the most striking feature was the excretion of considerable amounts of 5-hydroxytryptophan, as well as of 5-HT and 5-HIAA. The most likely explanation for this was that metastases existed in the kidney, and that the 5-hydroxytryptophan was formed in the argentaffin cells and excreted before all was metabolized to 5-HT. The existence of kidney metastases was confirmed clinically by intravenous pyelography, but unfortunately it was not possible to obtain permission to do a *post mortem* examination.

This case seemed to us to provide strongly suggestive evidence that formation of 5-hydroxytryptophan occurred in the argentaffin cells. We therefore turned our attention to perfusion of the blood vessels of the gut, as the major site of argentaffin cells is the intestinal mucosa. Unfortunately we were unable to establish conditions under which adequate oxygenation occurred. As the argentaffin cells, even in the tissues richest in them, are comparatively few in number and in that part of the tissue furthest from the larger tissue blood vessels, the occurrence of an oxidative reaction in these cells is likely to be particularly sensitive to oxygen supply.

The likelihood that formation of 5-hydroxytryptophan occurs in the argentaffin cells can also be supported on general grounds. The normal animal contains far more 5-hydroxytryptophan decarboxylase than is required to form 5-HT at the normal rate. The limiting factor is therefore availability of substrate. If, as far as 5-HT is concerned, argentaffin cells were concerned only with decarboxylation, the presence of a large extra number of cells as in metastasizing carcinoid would not be expected to alter 5-HT output appreciably. As 5-HT output is markedly raised, the increase in number of argentaffin cells is presumably accompanied by a corresponding increase in formation of the 5-hydroxytryptophan needed as substrate for the decarboxylase.

In view of the possible relation of 5-HT to brain function it is of interest that for many years some histologists (e.g., Masson and Berger, 1923 ; Masson, 1928) have considered that argentaffin cells bear a close relation to the nervous system. We consider that our results strongly suggest that the argentaffin cells are the main site of biosynthesis of 5-hydroxytryptophan. If this is the case the low concentration of argentaffin cells makes it seem likely that formation of 5-hydroxytryptophan will prove difficult to study by enzymic techniques except in tumour tissue.

### SUMMARY

1. The activity of 5-hydroxytryptophan decarboxylase in some tissues of the mouse and rat is reported.

2. Experiments with rat and mouse liver slices and homogenates and rat liver mitochondria showed no evidence for the conversion of tryptophan to 5-hydroxyindoles.

3. Perfusion of rat liver, under conditions in which the evidence suggested that liver function remained normal, gave no evidence for conversion of tryptophan to 5-hydroxyindoles.

4. These results, taken in conjunction with evidence published elsewhere, are considered to indicate that the major site of formation of 5-hydroxytryptophan is the argentaffin (enterochromaffin, Kultschitzky) cell system located principally in the intestinal mucosa.

This work was supported by a grant from the British Empire Cancer Campaign, to whom we are greatly indebted. We also thank the many individuals and firms (named in the experimental section) who have given us materials ; Dr. I Doniach for advice on perfusions ; Dr. A. G. E. Pearse for histological examinations ; Dr. F. A. Holton for advice on cell fractionation ; and Mr. D. Bush for assistance.

### REFERENCES

Asatoor, A. and Dalgliesh, C. E.—(1956) *J. chem. Soc.*, p. 2291.

Brauer, R. W., Leong, G. F., McElroy, R. F. and Holloway, R. J.—(1956) *Amer. J. Physiol.*, **184**, 593.

Brown, B. G. and Hey, P.—(1956) *Brit. J. Pharmacol.*, **11**, 58.

de Castro, F. T., Price, J. M. and Brown, R. R.—(1956) *J. Amer. chem. Soc.*, **78**, 2904.

Copenhaver, J. H. and Lardy, H. A.—(1952) *J. biol. Chem.*, **195**, 225.

Dalgliesh, C. E.—(1955) *Advanc. Protein Chem.*, **10**, 31.—(1956) *Biochem. J.*, **64**, 481.

*Idem* and Dutton, R. W.—(1956) *J. chem. Soc.*, p. 3748.—(1957) *Biochem. J.*, **65**, 21P.

*Idem* and Tabechian, H.—(1956) *Ibid.*, **62**, 625.

Erspamer, V.—(1954*a*) *Experientia*, **10**, 471.—(1954*b*) *R.C. sci. Farmitalia* **1**, 1.
Gaddum, J. H. and Giarman, N. J.—(1956) *Brit. J. Pharmacol.*, **11**, 88.
Gordon, A. H.—(1956) *Biochem. J.*, **64**, 58P.
Henriques, O. B., Henriques, S. B. and Neuberger, A.—(1955) *Ibid.*, **60**, 409.
Hier, S. W. and Bergeim, O.—(1946) *J. biol. Chem.*, **163**, 129.
Hogeboom, G. E.—(1955) 'Methods in Enzymology.' Vol. 1. New York (Academic Press), p. 16.
Humphrey, J. H. and Toh, C. C.—(1954) *J. Physiol.*, **124**, 300.
Laurence, D. J. R.—(1957) *Biochem. J.*, **65**, 27P.
Macfarlane, P. S., Dalgliesh, C. E., Dutton, R. W., Lennox, B., Nyhus, L. M. and Smith, A. N.—(1956) *Scot. med. J.*, **1**, 148.
McIlwain, H. and Buddle, H. L.—(1953) *Biochem. J.*, **53**, 412.
Masson, P.—(1928) *Amer. J. Path.* **4**, 181.
*Idem* and Berger, L.—(1923) *C.R. Acad. Sci., Paris*, **176**, 1748.
Miller, L. L., Bly, C. G., Watson, M. L. and Bale, W. F.—(1951) *J. exp. Med.*, **94**, 431.
Mitoma, C., Weissbach, H. and Udenfriend, S.—(1955) *Nature*, **175**, 994.
Price, J. B. and Dietrich, L. S.—(1956) *Fed. Proc.*, **15**, 330.
Sheffner, A. L. and Bergeim, O.—(1954) *Arch. Biochem. Biophys.*, **49**, 327.
Smith, A. N., Nyhus, L. M., Dalgliesh, C. E., Dutton, R. W., Lennox, B. and Macfarlane, P. S.—(1957) *Scot. med. J.* **2**, 24.
Thorson, A., Biörck, G., Björkman, G. and Waldenstrom, J.—(1954) *Amer. Heart J.*, **47**, 795.
Toh, C. C.—(1954) *J. Physiol.*, **126**, 248.
Udenfriend, S., Bogdanski, D. F. and Weissbach, H.—(1955) *Science*, **122**, 972.—(1956) *Fed. Proc.*, **15**, 493.
*Idem*, Clark, C. T. and Titus, E.—(1953*a*) *Ibid.*, **12**, 282.—(1953*b*) *J. Amer. chem. Soc.*, **75**, 501.
*Idem*, and Cooper, J. R.—(1952) *J. biol. Chem.*, **194**, 503.
*Idem*, Titus, E. and Weissbach, H.—(1955) *Ibid.*, **216**, 419.
*Idem*, Weissbach, H. and Clark, C. T.—(1955) *Ibid.*, **215**, 337.
Umbreit, W. W., Burris, R. H. and Stauffer, J. F.—(1949) 'Manometric Techniques and Tissue Metabolism', 2nd edn. Minneapolis (Burgess Publishing Co. ) p. 119.